# Redefining the pattern of age-prospective memory-paradox: new insights on age effects in lab-based, naturalistic, and self-assigned tasks

**DOI:** 10.1007/s00426-018-1140-2

**Published:** 2018-12-26

**Authors:** Katharina M. Schnitzspahn, Lia Kvavilashvili, Mareike Altgassen

**Affiliations:** 1 School of Psychology, University of Aberdeen, Aberdeen, AB24 3FX UK; 2 Department of Psychology, University of Hertfordshire, Hatfield, UK; 3 Donders Institute for Brain, Cognition and Behaviour, Radboud University Nijmegen, Nijmegen, The Netherlands

## Abstract

Prospective memory (PM) involves remembering intended actions in the future, such as posting a letter when seeing a post box (event-based PM) or making a phone call at 2:00 pm (time-based PM). Studies on aging and PM have often reported negative age effects in the laboratory, but positive age effects in naturalistic tasks outside the laboratory (the so-called age–PM-paradox). The present study re-examined this pattern of the paradox by studying, for the first time, age differences in time- and event-based PM in lab-based, experimenter-generated naturalistic and self-assigned real-life PM tasks within the same sample of young and older adults. Results showed that differential age effects in and outside the laboratory were qualified by the type of PM cue. While age-related deficits were obtained for laboratory event-based tasks, no age effect was obtained for naturalistic event-based PM. Age benefits in the field were only observed for naturalistic time-based tasks, but not for participants’ own self-assigned time-based tasks. These findings indicate that the age benefits for naturalistic PM tasks may have been overestimated due to the dominant use of experimenter-generated naturalistic time-based PM tasks in previous studies. Therefore, the precise pattern of the age–PM-paradox may need redefining as mostly consisting of negative age effects in lab-based PM tasks and mostly the absence of negative age effects (rather than age benefits) in naturalistic and self-assigned tasks outside the laboratory.

## Introduction

Remembering to take one’s medication, pay a bill on time or send a birthday card to a friend are all examples of prospective memory (PM) tasks, which involve self-initiated retrieval of intended actions at a specific moment in the future (Ellis & Kvavilashvili, 2000; Kliegel, McDaniel, & Einstein, 2007). They are often contrasted with retrospective memory tasks, which involve the externally prompted retrieval of past information such as the recall of previously studied words in a free recall test or someone’s name when meeting them for the second time. Another important feature of PM tasks, in addition to their self-initiated nature, is that they have to be performed when one is occupied with a competing activity at the same time, the so-called ongoing task (McDaniel & Einstein, 2000). PM tasks can be classified as event-based tasks, in which the execution of the intended action is initiated in response to a particular target event or cue (e.g., posting a letter when passing a post box), or time-based tasks, which require remembering to perform the intended action at a specific time or after a specified period of time has elapsed (e.g., making a phone call at 2:00 pm, taking the cake out of the oven after 40 min). PM has been identified as one of the most frequent everyday memory challenges (Kliegel & Martin, 2003), and given the impact that PM can have on older adults’ instrumental activities in everyday life (Woods, Weinborn, Velnoweth, Rooney, & Bucks, 2012), many studies have focused on the exploration of possible age effects on PM.

One of the most surprising and perplexing findings that have emerged from this research concerns a contrasting pattern of age-related PM performance in different task settings: While age-related deficits are often found in standard lab-based PM tasks, age-related benefits occur in naturalistic PM tasks, with older adults outperforming young participants (Kliegel, Rendell, & Altgassen, 2008; Phillips, Henry, & Martin 2008). Lab-based studies usually involve a dual-task paradigm consisting of an ongoing activity (e.g., lexical decision task) that needs to be interrupted to carry out an additional PM task (e.g., press a key when a particular word occurs or at a specific point in time during the ongoing task; Einstein & McDaniel, 1990). Typical naturalistic PM tasks comprise tasks such as mailing a postcard to the experimenter (e.g., Patton & Meit, 1993) or calling the experimenter at pre-defined times (e.g., Maylor, 1990). The everyday activities that participants are engaged in, when the right moment to perform the naturalistic PM task arises (e.g., walking down the street or reading a newspaper, respectively), represent ongoing activities equivalent to the ongoing task in the laboratory. As in the laboratory, these ongoing activities may vary in the amount of attentional resources required.

The pattern of differential age effects observed in PM as a function of the task setting has been called the age–PM-paradox (Rendell & Craik, 2000), and it currently constitutes “one of the most important puzzles in the study of cognitive aging” (p. 3, Verhaegen, Martin, & Sedek, 2012). The positive age effects outside the laboratory are puzzling, because they are counterintuitive and contradict theories of cognitive aging, which would at best predict absence of age effects for those naturalistic PM tasks that may be mediated by automatic retrieval processes due to strong external environmental support and/or relying on some compensatory strategies (e.g., Craik, 1986; Grady & Craik, 2000; Hasher & Zacks, 1988). Similarly, positive age effects would not be predicted by the influential multiprocess theory of PM, which assumes that PM performance can be mediated by both automatic and strategic monitoring processes, depending on the context and task requirements (McDaniel & Einstein, 2000, 2007; Scullin, McDaniel, & Shelton, 2013). Thus, although many short-delay laboratory PM tasks require strategic monitoring for the target events or times, which may be accounting for negative age effects, in everyday situations such constant monitoring would be unrealistic and unnecessary over longer delay periods of hours or days, and people would be more likely to rely on spontaneous retrieval processes (cf. Scullin et al., 2013). If this was the case (for initial evidence see Ellis & Nimmo-Smith, 1993; Kvavilashvili & Fisher, 2007; Sellen, Louie, Harris, & Wilkins, 1997), then the multiprocess theory would predict absence of age effects in everyday PM tasks, but not positive age effects reported by naturalistic studies (Devolder, Brigham, & Pressley, 1990; Moscovitch, 2008; Rendell & Thomson, 1993, 1999).

Research on the age–PM-paradox is important not only theoretically (as it can enhance or even change our understanding of cognitive aging), but also because it raises the intriguing possibility that PM performance may be one of the few cognitive abilities that may be spared by negative effects of aging in everyday life (Henry, MacLeod, Phillips, & Crawford, 2004; Phillips et al., 2008). Real-life PM tasks are intentions that participants set up themselves without interference of an experimenter (i.e., actual intentions participants form, try to remember and fulfill at specific times or events). Most studies on the age-PM-paradox have used naturalistic tasks that were more or less artificial and externally assigned by an experimenter. It is therefore essential to determine whether findings from studies with experimenter assigned ‘naturalistic’ PM tasks generalize to remembering actual real-life PM tasks formed by participants themselves in the course of their daily life.

Although the age–PM-paradox has been confirmed by two meta-analyses of earlier studies (Henry et al., 2004; Uttl, 2008), research in this area is still in its infancy, despite its huge theoretical and practical implications. First, the current formulation of the paradox has been based primarily on separate studies that have tested PM in the laboratory (using predominantly event-based tasks), or in everyday life using primarily naturalistic time-based tasks (e.g., see Henry et al., 2004). Second, only a handful of studies have tested the same samples of young and older adults in and outside the laboratory (Kvavilashvili, Cockburn, & Kornbrot, 2013; Niedźwieńska & Barzykowski, 2012; Rendell & Craik, 2000,1; Rendell & Thomson, 1999; Schnitzspahn, Ihle, Henry, Rendell, & Kliegel, 2011), and out of these, only two have tested both time-and-event-based tasks within the single study (Niedźwieńska & Barzykowski, 2012; Rendell & Craik, 2000). Third, four studies investigated age effects on real-life PM tasks formed by participants themselves, but they did not assess participants’ PM performance in and outside the laboratory with standard experimenter-assigned laboratory and naturalistic tasks to provide comparisons across settings and types of tasks (Freeman & Ellis, 2003; Ihle, Schnitzspahn, Rendell, Luong, & Kliegel, 2012; Niedźwieńska, Janik, & Jarczynska, 2013; Schnitzspahn et al., 2016). Finally, and perhaps most importantly, while positive age effects have been relatively consistently obtained for time-based tasks outside the laboratory, inconsistent patterns have been obtained in those few studies that have used naturalistic event-based tasks (see details below). Taken together, the nature of the age–PM-paradox is far from clear and calls for more focussed and systematic investigation.

The study of the age–PM-paradox is becoming especially important in the light of accumulating evidence which shows that the typical pattern often cited in the literature, with negative age effects in the lab and positive age effects outside the lab, may not be entirely accurate and may need careful re-examination using a within-subjects design. Consequently, the primary goal of the present investigation was to make significant advances in research on the age–PM-paradox by establishing the precise pattern of age effects for all three types of tasks (laboratory, naturalistic experimenter-assigned, and real-life self-assigned PM tasks) within the same sample of young and older adults, while manipulating the type of PM cue (time vs. event) in and outside the laboratory. None of the previous studies have tested all three types of PM tasks within one experiment and only two studies compared event-based and time-based PM in and outside the laboratory within one sample of young and older adults with conflicting results concerning the effects of age on naturalistic event-based tasks (Niedźwieńska & Barzykowski, 2012; Rendell & Craik, 2000).

## The age–PM-paradox for time-based tasks

Time-based PM tasks have been studied in the laboratory either with computerised ongoing tasks where participants have to remember to press a key once every few minutes (e.g., 2–5 min, depending on the study; Einstein, McDaniel, Richardson, Guynn, & Cunfer, 1995; Park, Hertzog, Kidder, Morrell, & Mayhorn, 1997) or by asking participants to note when a particular amount of time has elapsed from the start of the session (e.g., 10 and 20 min) involving several different cognitive tasks (both paper-and-pencil and computerised). In the former, participants can check the elapsed time by pressing another computer key, which brings a digital clock onto the screen for few seconds, and in the latter, participants often can check a real clock on the wall or a table nearby. Performance is usually measured by the proportion of on-time responses, with varying time-windows used in different studies for counting a response as on time (from few seconds to 60 s).

Because participants have to remember to check the clock themselves without the help of any external cues, the widely held theoretical view is that remembering time-based tasks is more effortful than remembering event-based tasks (Einstein et al., 1995; McDaniel & Einstein, 2007). Therefore, larger age effects are expected for time- than event-based tasks, and overall, negative age effects on-time-based PM tasks have indeed been demonstrated in several studies (e.g., Einstein et al., 1995; Kvavilashvili, Kornbrot, Mash, Cockburn, & Milne, 2009; Park et al., 1997; Rose, Rendell, McDaniel, Aberle, & Kliegel, 2010). However, there are also studies that have failed to obtain negative age effects on laboratory time-based tasks (e.g., d’Ydewalle, Luwel, & Brunfaut, 1999; Logie, Maylor, Della Salla, & Smith, 2004; Niedźwieńska & Barzykowski, 2012), which suggests that the current theoretical understanding of time-based PM, as based primarily on effortful monitoring processes, may be oversimplified or incorrect (e.g., Kvavilashvili & Fisher, 2007; for more detailed discussion of possible reasons for the absence of age effects in some laboratory tasks of time-based PM, see “Discussion” section).

In naturalistic studies of time-based PM, participants are usually asked to remember to carry out simple tasks such as sending a postcard, a text message, making a phone call or operating an electronic device at regular intervals (sometimes several times a day) over a time period of several days. In contrast to negative age effects that are often obtained in the laboratory, almost all naturalistic studies of time-based PM have reported significant positive age effects (Devolder et al., 1990; Moscovitch, 1982; Niedźwieńska & Barzykowski, 2012; Patton & Meit, 1993; Rendell & Craik, 2000; Rendell & Thomson, 1993, 1999; Schnitzspahn et al., 2011), a pattern that was also confirmed by the Henry et al. (2004) meta-analysis.

Three of these studies have used the same participants in and outside the laboratory (Niedźwieńska & Barzykowski, 2012; Rendell & Thomson, 1999; Schnitzspahn et al., 2011). The initial study by Rendell and Thomson (1999) with large samples of young, young–old and old–old participants used a variety of time schedules (some of them quite complex), but these manipulations did not affect the results (both groups of older adults outperformed the young). One potential limitation of the study by Rendell and Thomson (1999) was that a proportion of younger adults was admitting being unable to operate a device due to occasionally leaving it behind, which did not happen to older adults. However, more recent studies, which have used participants’ own mobile phones (which people rarely leave behind) still fully confirmed the validity of initial findings and older adults’ superiority in carrying out time-based PM tasks (Niedźwieńska & Barzykowski, 2012; Schnitzspahn et al., 2011).

Nevertheless, some naturalistic studies of time-based PM have failed to obtain age benefits and have instead reported no reliable age effects. Closer examination of these studies indicates that they involved single, one-off tasks rather than carrying out the same action many times over several days. For example, in a study by Kvavilashvili and Fisher (2007), young and older participants met with a researcher on Monday morning and had to remember to make a single phone call to the researcher at their chosen time on Sunday (after 6 days). Similarly, in a study by West (1988, study 1), participants had to send a postcard 2 days after an interview with a researcher. Arguably, such tasks are more similar to real-life time-based PM tasks, which rarely involve carrying out the same action at a particular time over many days (except, for instance, when on antibiotic treatment).

Taken together, these initial findings suggest that the positive age effects in naturalistic time-based tasks may have been, at least partly, due to using a particular type of time-based task, which involves repeatedly carrying out the same action at specific times over a period of time. This conjecture will be investigated in the present study by asking participants to carry out only one-off time-based tasks rather than repeated time-based tasks as in most previous studies.

## The age–PM-paradox for event-based tasks

Although most laboratory research on PM is being conducted on event-based PM, there are only a handful of studies that have investigated event-based PM outside the laboratory, probably due to difficulties in designing naturalistic event-based tasks (cf. Phillips et al., 2008). Moreover, so far only Rendell and Craik (2000) have reported positive age effects for naturalistic event-based tasks, while the three studies that assessed the paradox for event-based tasks in one sample of young and older adults resulted in either no age effects (Kvavilashvili et al., 2013; Niedźwieńska & Barzykowski, 2012) or a negative age effect with young adults performing better than old–old participants (Dobbs & Rule, 1987; see also Bailey, Henry, Rendell, Phillips, & Kliegel, 2010). In addition, while participants in the Niedźwieńska and Barzykowski (2012) study had to remember to execute an event-based task on several occasions (as in most naturalistic time-based PM tasks), participants in the Kvavilashvili et al. (2013) study had to complete only a single task by putting their name and the date on a front page of a questionnaire after filling it in at home and posting it back to the researcher (a task used originally by Dobbs & Rule, 1987). As no age effects were obtained in both studies, it is less likely that the absence of the positive age effect depended on the nature of the task (single-occasion vs. multiple-occasion).

Consequently, available evidence on naturalistic event-based PM is mixed and does not support a current formulation of the age–PM-paradox, which emphasises the presence of positive age effects for naturalistic PM tasks outside the laboratory. More balanced conclusions could be achieved if studies on the paradox started including event-based tasks into their design. Therefore, in the present study, participants had to carry out two different naturalistic event-based tasks after completing the laboratory session.

## Effects of age on real-life self-assigned PM tasks

Four studies have investigated age differences in actual real-life PM behaviour by asking participants to list their intended PM tasks for a given time period (e.g., the next day or week), and indicate which tasks were completed and forgotten (or re-prioritised) at the end of this time period (Freeman & Ellis, 2003; Ihle et al., 2012; Niedźwieńska et al., 2013; Schnitzspahn et al., 2016). Although these studies have reported positive age effects in real-life PM task completion, they have also shown that on average, older adults gave higher ratings of intention importance (Ihle et al., 2012; Niedźwieńska et al., 2013; Schnitzspahn et al., 2016) and displayed the tendency to form well-planned intentions with more contextual details than young adults (Niedźwieńska et al., 2013). Nevertheless, importance and intention specificity cannot entirely explain the positive age effects obtained in these studies. Although positive age effects indeed disappeared when young and older participants’ PM performance was compared for important or well-defined intentions (based on participants’ own ratings), they were still present for less important (Ihle et al., 2012; Niedźwieńska et al., 2013) and less specific intentions (Niedźwieńska et al., 2013). Overall, these findings suggest that possible mediators of age differences exist and that in everyday life, young adults can perform as well as older adults under certain conditions (see also Aberle, Rendell, Rose, Kliegel, & McDaniel, 2010). Therefore, more evidence is needed before one can make strong claims that, in general, older adults outperform young adults when remembering their own rather than experimenter-generated PM tasks in everyday life.

## The present study

The literature reviewed in previous sections indicates that despite initial progress in demonstrating the age–PM-paradox, there are several unanswered questions not only in terms of the exact pattern of the paradox for laboratory and naturalistic time-and event-based PM tasks, but also in terms of whether positive age effects on naturalistic time-based tasks generalize to participants’ own self-assigned real-life intentions. Therefore, the aim of the present study was to clarify the precise pattern of age-related decrements in the laboratory and age-related benefits in both naturalistic and real-life self-assigned PM tasks as a function of PM cue type (time vs. event) within one sample of young and older participants.

In the laboratory, participants were given two different event-based tasks and one time-based task while being engaged in completing several different cognitive tasks and questionnaires. In everyday life, participants had to remember to carry out two single event-based tasks, and two different time-based tasks (text message and phone call), which varied in terms of time delay (1-day delay vs. 3-day delay). These single-occasion rather than repetitive (multiple occasion) time-based tasks were chosen to examine the idea that positive age effects may be less likely to occur with such single-occasion tasks (cf. Kvavilashvili & Fisher, 2007). In addition, participants were asked to list their own PM tasks that they intended to perform in the next few days, and later their performance success on these tasks was assessed. To explore if the type of cue in self-assigned PM tasks showed the same pattern of age effects as in naturalistic tasks, all reported intentions were categorized post-hoc, based on participants’ indication of a time or event related to the intention fulfillment.

The main prediction was that the standard pattern of the age–PM-paradox would be obtained for time-based but not for event-based tasks. While in the laboratory, negative age effects were predicted, outside the laboratory positive age effects were expected only for naturalistic time-based but not for event-based tasks. However, if positive age effects on-time-based tasks in previous research were due to using repetitive rather than single-occasion (one-off) time-based tasks, then no positive age effect would be expected for single-occasion-time-based tasks used in the present study. In relation to self-assigned PM tasks, it was expected that the pattern of findings would mimic the one obtained for naturalistic PM tasks with no age effects for event- but positive age effects for time-based tasks.

In addition to studying the pattern of the age–PM-paradox, we also examined two variables that have been often discussed in the literature as potentially important for explaining positive age effects outside the laboratory (Phillips et al., 2008). First, participants had to rate the importance of naturalistic PM tasks as well as of the self-assigned PM tasks. Perceived task importance has been suggested as one key motivational variable that can increase PM, and potentially explain age benefits in everyday life by differences in perceived importance of intentions between young and older adults (Phillips et al., 2008). If this was the case, then older adults would rate PM tasks for which age benefits were expected in the present study (i.e., time-based naturalistic and self-assigned tasks) as more important than young adults (cf. Ihle et al., 2012; Niedźwieńska et al., 2013; Schnitzspahn et al., 2016). Second, participants also reported whether they had used any reminders or strategies to carry out PM tasks in everyday life. One of the most popular explanations of the age–PM-paradox in the literature is that older adults are very good at using external reminders in everyday life, while they do not have this opportunity in lab-based tasks (Phillips et al., 2008). In the present study, we focused on possible differences in the use of reminders in naturalistic time- and event-based PM tasks to test the hypothesis that the expected age benefits in time-based tasks could be explained by a higher number of reminders used by older adults for this specific task type.

## Method

### Participants

The sample included 53 adults, 31 young (*M*_age_ = 23.71 years, SD = 3.07 years, age range 20–29 years; 12 men) and 22 older adults (*M*_age_ = 67.09 years, SD = 4.66 years, age range 60–75 years; 12 men). All young adults were undergraduate students from the local university who participated in exchange for partial course credit. All older adults were volunteers who were recruited using the participant pool from the developmental psychology chair in Dresden. Exclusion criteria were current physical and mental health and sensory problems including hearing or vision impairments. Age groups did not differ with respect to gender distribution, *χ*^2^(*df* = 1) = 1.30, *p* = .28, or the mean number of years in education, *F*(1, 51) = .31, *p* = .58, $\eta _{{\text{p}}}^{2}$ = .006 (Young *M* = 12.45, SD = 1.29; Old *M* = 12.23, SD = 1.63).

Typical age-related patterns were obtained in relation to general cognitive abilities and standard episodic memory tests (see Table 1). While older adults attained significantly higher scores than young adults on a German vocabulary test (MWT-B; Lehrl, 2005) measuring crystallized intelligence, young adults outperformed older adults on the Digit-Symbol-subtask of the Wechsler Adult Intelligence Scale (WAIS-IV; Wechsler, 2008) measuring fluid intelligence. In addition, young adults also outperformed older adults in immediate and delayed retrospective memory recall (measured with the Verbal Paired Associates subtask from the Wechsler Memory Scale revised; WMS-R, Wechsler & Härting, 2000). Similarly, there was a trend for better performance in young compared to older adults in immediate and delayed performance in the logical memory subtask from the WMS-R.

**Table 1:** Mean performance scores (standard deviations) on cognitive tasks as a function of age (young vs. old) and results of one-way ANOVAs on these means

Cognitive task	Age group				
	Young	Old	*F* (1, 51)	*p*	$\eta _{{\text{p}}}^{2}$
	*M* (SD)	*M* (SD)			
Vocabulary	30.71 (2.60)	33.50 (1.95)	18.13	< .001	.26
Digit-symbol	84.77 (11.45)	62.59 (11.67)	23.80	< .001	.48
Verbal paired associates	29.84 (1.59)	27.05 (3.48)	15.46	< .001	.23
Logical memory	56.74 (15.77)	49.09 (12.60)	3.56	.065	.07

## Materials

### Lab-based PM tasks

#### Event-based PM

Two tasks were used to measure event-based PM in the laboratory. In the pencil task, adapted from Dobbs and Rule (1987), participants were asked to repeat the words “blue pen” whenever the experimenter used these words during the testing session. This occurred on three occasions when participants were asked to fill in a questionnaire or complete a test with the blue pen, which was on the desk in front of the participant next to another pen of a different color. For the token task, adapted from Zeintl, Kliegel, and Hofer (2007), participants were asked to take a token out of a drawer and give it to the experimenter whenever the experimenter started the instruction of a new task with the sentence, “The next task concerns memory”. During the testing session, this particular sentence was clearly articulated by the experimenter on three occasions. No potentially misleading lure phrases were given. The drawer containing the tokens was part of a storage container placed underneath the desk. The number of tokens in the drawer was higher than the number of cues.

#### Time-based PM

A modified version of the stop-clock task was used to measure time-based PM in the laboratory (Rendell & Thomson, 1999). Participants were asked to remember to tell the experimenter when 10 and 20 min had passed immediately after the experimenter had finished instructions for all the laboratory PM tasks. A small table clock was placed behind the participant to enable them to monitor the elapsed time by turning around (cf. Harris & Wilkins, 1982). The clock was placed in a way that allowed the experimenter to easily monitor it and notice the start time. In line with previous studies, a time-window of 10 s before and after the target time was used to classify responses as correct PM answers (e.g., Kliegel, Martin, McDaniel, & Einstein, 2001; Rendell & Thomson, 1999).

### Naturalistic experimenter-assigned PM tasks

#### Event-based PM

Participants were instructed to remember to send a text message to the experimenter when they saw a public transport bus for the first time on the day following the laboratory session, reporting the bus line and the participant code. Responses were classified as correct when the text message was received on the correct day containing an existing bus number and the participant code. As a second task, participants were given a postcard at the end of the laboratory session and asked to remember to send it back to the experimenter 2 days after the session when they passed a post box (Patton & Meit, 1993). They were asked to write down their participant code and the date on which they put the card into the post box before sending it off. Responses were classified as correct if the card arrived at the University with the correct date and code written on it. Further, the postmark was checked and had to be from the agreed date or the following weekday.

#### Time-based PM

In the 1-day delay time-based naturalistic task, participants were asked during the laboratory session to send a text message containing their participant code to the experimenter at an agreed time on the following day. At the end of a phone call 3 days later, the experimenter and the participant agreed on a new time for a second text message the day after.

In the 3-day delay time-based naturalistic task, participants were instructed to remember to call the experimenter 3 days after the laboratory session at a specific time. At the end of this phone call, the experimenter and the participant agreed on a new time for a second telephone call 3 days later. Calls and messages up to 10 min before or after the agreed times were classified as correct PM answers.

### Real-life self-assigned PM tasks

At the end of the laboratory testing session, participants were asked to report up to seven intended activities that they had planned for the following 3 days. It was stressed that participants should not report routine tasks such as making one’s bed in the morning, but instead currently intended actions (e.g., calling the doctor to make an appointment). For each nominated plan, they were also given an option to indicate a particular date or time that they had in mind for carrying out the task. Further, they had to rate how important the intention was on a three-point Likert-scale (1 = less important, 2 = important, 3 = very important). Participants were informed that the aim of the study was to explore natural everyday memory behavior and that it was very important that they behaved in their usual manner, without trying harder than usual to remember their planned activities.

During the first telephone call 3 days after the laboratory session, participants were asked to recall all planned activities and indicate which of them they had completed successfully and if they used a reminder. Participants were then asked to nominate up to seven new intended activities for the next 3 days. Their correct recall and successful performance was recorded by the experimenter during a second phone call 3 days later. For statistical analyses, real-life PM performance was aggregated across the 6 days of assessment. Percentages of correctly fulfilled intentions were used to account for differences in the overall number of planned intentions between participants. Reminder use and intention importance were only assessed for the first naturalistic and self-assigned PM tasks (study days 1–3). For statistical analyses, reminder use was calculated as the percentage of tasks for which participants reported to have used reminders. Importance ratings were added and divided by the number of tasks/intentions to obtain the average importance rating for each task type.

Given that the main goal of the present study was to test PM age differences as a function of the cue type (i.e., time- vs. event-based) across different settings, participants’ descriptions of intended activities and information provided on dates/times for the execution of self-assigned PM tasks were used to categorize these intentions into time-based (i.e., when the participant indicated only the date and/or time) and event-based PM tasks (i.e., when the participant stated an event irrespective whether the date or time for the intention fulfillment was indicated). This classification was based on the assumption that in everyday event-based tasks, participants may often have some idea about the time frame in which the event may be encountered. Surprisingly, almost all recorded self-assigned PM tasks (99%) mentioned a date and/or time, but only one nominated intention description specified an event for the intended action (i.e., passing a message to a family member), which made a distinction into time- and event-based PM tasks not feasible. However, a closer look at the time-based self-assigned tasks showed clear differences in the specificity of the time period for carrying out PM tasks. In particular, four different categories could be identified: (1) exact date and time (e.g., Monday at 10 am), (2) a time-window (e.g., Wednesday morning), (3) an entire day (e.g., Thursday) or (4) a deadline (e.g., before Sunday).^2^ The absolute numbers of self-assigned PM tasks within each category and the percentage of their correct fulfillment were used in the statistical analyses.

## Procedure

All participants started with an individual session in the laboratory. They were informed that the study goal was to examine verbal abilities, memory, information processing and personality. After signing consent forms, they received instructions for all three lab-based PM tasks. At first, the event-based pencil and the token tasks were explained and participants were asked to repeat instructions in their own words to ensure correct understanding. After the experimenter was satisfied that the participant had understood and encoded the pencil and the token tasks, the time-based stop-clock task was introduced. Again, instructions had to be repeated by the participants and the experimenter informed them that the time started running by pointing to the table clock behind them.

All PM tasks were embedded in a series of paper–pencil tasks and questionnaires (see overview of the procedure in Table 2) that served as ongoing activities. The event-based PM cue sentences were part of the task instruction or the request to fill in a questionnaire, respectively. Specifically, after repeating instructions for the stop-clock task, the testing session began and participants were asked to fill in the socio-demographic questionnaire using the ‘blue pen’, which was the first cue for the pencil PM task (i.e., repeating the words ‘blue pen’ when hearing these words from the experimenter). Then, participants performed the logical memory subtask from the WMS-R that the experimenter introduced by saying out loud “The next task concerns memory”, which was the first cue to perform the token PM task (i.e., giving a token to the experimenter). Following the logical memory subtask, participants performed the Verbal Paired Associates subtask from the WMS-R. Participants continued with the Digit-Symbol-Task, which was introduced by the experimenter by saying “Please use the blue pen to work on the next task”, which was the second cue for the pencil PM task. Afterwards, participants worked on the vocabulary test. Then they were asked to fill in the conscientiousness subscale of the German translation (Borkenau & Ostendorf, 1993) of the NEO Five-Factor Inventory (NEO-FFI; Costa & McCrae, 1992). The experimenter introduced the next task with the cue sentence for the token task (i.e., “the next task concerns memory”) and asked the participants to fill in the Prospective and Retrospective Memory Questionnaire (PRMQ, Crawford, Smith, Maylor, Della Sala, & Logie, 2003). Participants had to perform the delayed recall of the two WMS-R subtasks afterwards. The delayed recall was once more introduced with the cue sentence for the third token task. Finally, participants were asked about their planned activities for the next 3 days. The experimenter wrote down the intentions and related dates or times for their planned fulfillment if specified by the participant. The participants then received instructions for the naturalistic PM tasks, which they had to repeat in their own words to ensure correct understanding. In addition, they had to indicate how important these tasks were to them using a three-point Likert-scale (1 = less important, 2 = important, 3 = very important). The experimenter asked the participant to return the ‘blue pen’ (i.e., third cue for the pencil PM task) before they left. The whole session lasted approximately 45 min. Although the testing session was self-paced, for the majority of participants the opportunity to perform the first time-based stop-clock task (i.e., inform the experimenter that 10 min had expired) occurred during or after the logical memory subtask (see Table 2). Similarly, the second target time of 20 min occurred during or after the vocabulary test.

**Table 2:**
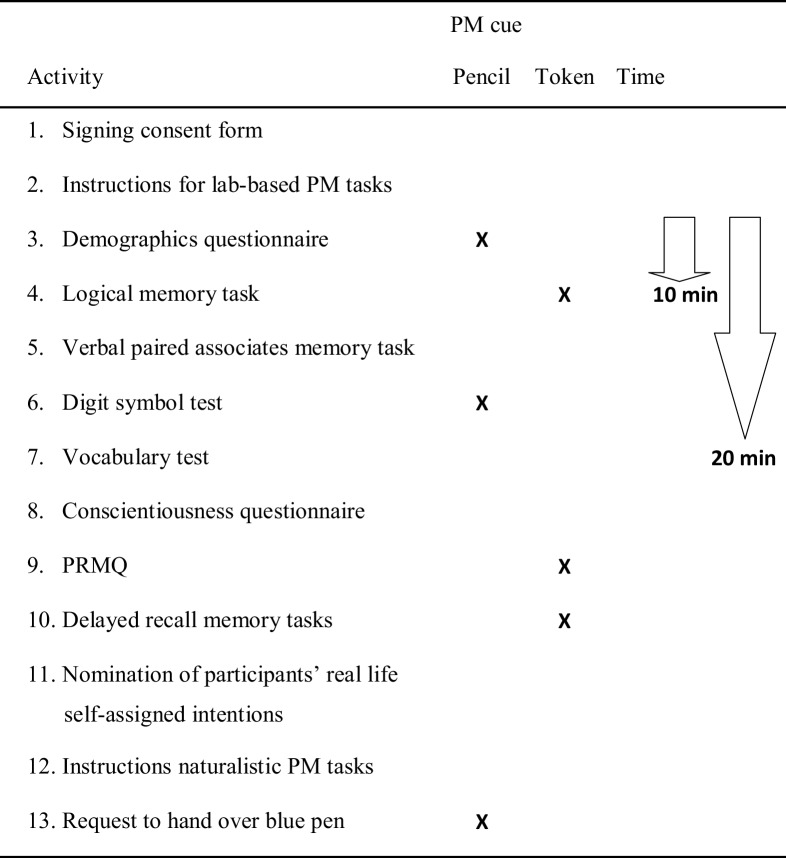
Overview of lab-based testing session listing all performed activities and indicating which activity instructions comprised a PM target cue (for the pencil or token task, respectively) and when the time-based PM task had to be performed

In the pencil task, participants were asked to repeat the words “blue pen” whenever the experimenter used them. In the token task, participants were asked to take a token out of a drawer whenever the instruction of a new task started with the sentence “The next task concerns memory”. In the time-based task, participants were asked to remember to tell the experimenter when 10 and 20 min had passed

**Table 3:** Overview of all the naturalistic tasks that participants had to complete over the 6 days following the lab-based testing session

Day	Task type	Activity
1	Time-based (1-day delay) Event-based	Send SMS with ID at arranged time Send SMS with ID and bus number when seeing a bus
2	Event-based	Send postcard with ID and date when passing post box
3	Time-based (3-day delay)	Call experimenter at agreed time
4	Time-based (1-day delay)	Send SMS with ID at arranged time
6	Time-based (3-day delay)	Call experimenter at agreed time

On the first day after the experimental session, participants had to remember two naturalistic PM tasks, one 1-day delay time-based (send a text message at a particular time) and one event-based (send a text message when seeing a bus). On the second day, participants had to remember to send a postcard when seeing a post box (event-based PM task). On the third day after the session, participants had to remember the 3-day delay time-based task to call the experimenter at the agreed time. If the participant forgot to call, the experimenter called him/her to ask if they used reminders for the naturalistic PM tasks and to assess remembering and performance of self-generated real-life PM tasks. Specifically, participants were asked to recall their planned activities. If one or several initially intended actions were not mentioned by the participant, the experimenter gave a general prompt by saying that these were not all planned activities. If the participant still could not recall all intentions, the experimenter told him/her which ones were missing. Then the experimenter repeated the whole list and the participant indicated if each intention was performed as planned. If this was not the case, reasons for non-fulfillment were assessed. Participants were also asked for each intention if they used a reminder. After that the experimenter asked for the planned activities for the next 3 days and also gave instructions for two new naturalistic time-based PM tasks. The 1-day delay task involved sending a text message on the day following the phone conversation, and the 3-day delay task involved calling the experimenter 3 days later (see overview of all naturalistic PM tasks in Table 3). If the participant did not call on the third day at the agreed time, the experimenter called herself and assessed the remembering and performance of participants’ own self-assigned real-life PM tasks as described above.

## Results

### PM performance as a function of age group, PM task and cue type

Participants completed several time- and event-based PM tasks in and outside the laboratory. To assess the overall pattern of the paradox for the lab-based and naturalistic PM tasks as a function of PM cue (event vs. time), we obtained single measures of laboratory and naturalistic time- and event-based PM by averaging scores over the observations and tasks. For example, the score for the laboratory time-based task was based on the proportion of on-time responses after 10 and 20 min. In the naturalistic event-based task, it was based on the proportion of correct responses in the bus and the post box tasks (see Table 4).^3^ To derive a single measure for laboratory event-based PM and naturalistic time-based PM, we averaged scores across the pen and token tasks and 1-day and 3-day delay tasks, respectively. This was justified by the results of two separate 2 (age group) × 2 (type of task) mixed ANOVAs on laboratory event-based and naturalistic time-based PM tasks, respectively, that did not result in significant main effects of task type (e.g., pen vs. token) or task type by age group interactions (all *F*s < 2.68).

**Table 4:** Mean percentages of correct PM responses (standard deviations) in young and older adults as a function of laboratory PM task (pencil, token, and stop-clock) and naturalistic PM task (event-based bus and post box, time-based 1-day delay and 3-day delay)

PM task	Age group	
	Young	Old
	*M* (SD)	*M* (SD)
Laboratory tasks		
Event-based (pencil)	91.40 (17.14)	54.55 (34.95)
Event-based (token)	88.17 (23.65)	56.06 (36.20)
Time-based (stop-clock)	75.81 (36.22)	77.27 (36.93)
Naturalistic tasks		
Event-based (bus, post box)	82.26 (24.32)	93.18 (17.56)
Time-based 1-day delay	56.45 (40.30)	81.82 (32.90)
Time-based 3-day delay	69.35 (33.36)	79.55 (29.52)

**Fig. 1:**
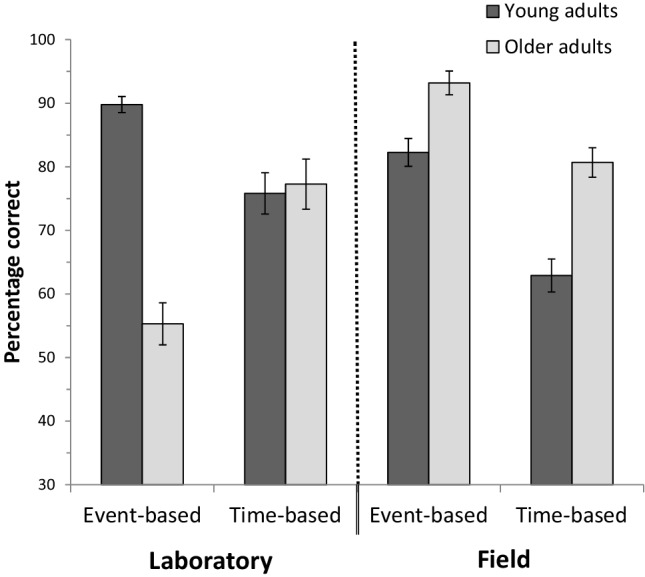
PM performance (percentage of correct PM responses) in both age groups as a function of task setting and PM cue type. Error bars represent the standard error (SE)

A 2 (age group: young vs. old) × 2 (PM task: naturalistic vs. lab-based) × 2 (PM target cue: event- vs. time-based) mixed-model ANOVA was then performed on the mean proportions of correct PM responses (see Fig. 1). Results showed that the main effects of age and PM task were not significant (both *p*s ≥ .217), but the main effect of PM target cue was approaching significance with medium size effect, *F* (1, 51) = 3.35, *p* = .073, $\eta _{{\text{p}}}^{2}$ = .06. However, all two-way interactions between PM target cue and PM task [*F* (1, 51) = 7.39, *p* = .009, $\eta _{{\text{p}}}^{2}$ = .13], PM target cue and age [*F* (1, 51) = 10.76, *p* = .002, $\eta _{{\text{p}}}^{2}$ = .17] and age and PM task [*F* (1, 51) = 13.68, *p* = .001, $\eta _{{\text{p}}}^{2}$ = .21], were significant. Importantly, in line with predictions, these interactions were qualified by a marginally significant three-way interaction of medium effect size, *F* (1, 51) = 3.94, *p* = .053, $\eta _{{\text{p}}}^{2}$ = .07.

Exploring the triple interaction, several follow-up analyses were conducted separately for the two settings. In the laboratory, young adults outperformed older adults in the event-based PM tasks, *F*(1, 51) = 29.84, *p* < .001, $\eta _{{\text{p}}}^{2}$ = .37, while performance in the time-based tasks did not differ between age groups, *F* < 1. In contrast, in the naturalistic PM tasks, older adults outperformed young adults in the time-based tasks, *F*(1, 51) = 5.97, *p* = .018, $\eta _{{\text{p}}}^{2}$ = .11, but the age effect was not statistically significant for the event-based tasks, *F*(1, 51) = 3.23, *p* = .078, $\eta _{{\text{p}}}^{2}$ = .06.

Further follow-up analyses were performed separately for the two age groups. Within the young adults, PM performance was significantly better for event-based than time-based cues (see Fig. 1). This finding was true in the laboratory, *F*(1, 30) = 4.67, *p* = .039, $\eta _{{\text{p}}}^{2}$ = .14, and in the field, *F*(1, 30) = 12.27, *p* = .001, $\eta _{{\text{p}}}^{2}$ = .29. There were no main effects of setting for event- or time-based PM tasks, both *p*s ≥ .174.

Within the older adults, PM performance was better for time-based than event-based tasks in the laboratory, *F*(1, 21) = 4.76, *p* = .041, $\eta _{{\text{p}}}^{2}$ = .19. The opposite pattern emerged in the field with better performance in event-based than time-based tasks, *F*(1, 21) = 5.92, *p* = .024, $\eta _{{\text{p}}}^{2}$ = .22. In addition, performance was significantly better in the event-based PM tasks in the field compared to performance in the lab-based tasks, *F*(1, 21) = 24.76, *p* < .001, $\eta _{{\text{p}}}^{2}$ = .54, while performance across settings (laboratory vs. field) was comparable for time-based tasks, *p* = .650.

To test the robustness of this pattern of results, we conducted an additional nonparametric permutation test for the main analysis on PM performance as a function of age group, PM task and PM target cue. Each permutation test was based on 1000 random permutations of the original data (Good, 2005). The results of this analysis converged with the pattern of findings obtained in the parametric ANOVA (see Table 5).

**Table 5:** Results of nonparametric permutation test on PM performance as a function of age group, PM task and PM target cue

Effect	*F*	*p*
Age group	.07	.795
PM task	.32	.580
PM target cue	4.93	.026
Age group × cue	10.76	.002
Age group × task	13.68	< .001
Cue × task	5.52	.021
Age group × cue × task	3.94	.051

### Real-life self-assigned PM as a function of age and temporal specificity

The number of planned activities in the first 3 days and the subsequent 3 days were added up for each participant. There was a trend for a higher number of self-assigned intentions across the 6 days in young (*M* = 10.36; SD = 1.42) compared to older adults (*M* = 9.70; SD = .98), *F* (1, 46) = 3.19, *p* = .081, $\eta _{{\text{p}}}^{2}$ = .07. Participants reported intentions such as arranging a foot care appointment, buying a bargain offer, sorting and putting holiday pictures in the photo album or preparing a specific meeting.

We also examined the number of planned activities as a function of age and temporal specificity by conducting a 2 (age group: young vs. old) × 4 (specificity: exact date and time vs. time-window vs. specific day vs. deadline) mixed-model ANOVA with the repeated measures on the second factor. Results showed a significant main effect of specificity, *F* (3, 129) = 30.50, *p* < .001, $\eta _{{\text{p}}}^{2}$ = .42. The main effect of age and the specificity by age interaction was not significant, *F*s < *1*. Both young and older adults mainly indicated to have set themselves intentions with certain time-windows (e.g., Monday evening) rather than intentions with the precise time and date (e.g., 8:00 pm on Monday) or less specific intentions involving a particular day or a deadline (see Table 6).

**Table 6:** Mean number and range of planned intentions (standard deviations in brackets) and percentages of fulfilled self-assigned real-life PM tasks in both age groups as a function of cue specificity

	Young adults				Older adults			
	Date and time	Time-window	Specific day	Deadline	Date and time	Time-window	Specific day	Deadline
Number of intentions	1.74 (2.14)	5.81 (3.70)	1.33 (1.96)	1.19 (2.30)	.83 (1.34)	6.33 (2.83)	2.11 (2.40)	1.00 (2.11)
Range	0–7	0–13	0–8	0–9	0–3	1–10	0–9	0–7
Percentage of fulfilled Intentions	75.34 (27.07) (*n* = 14)	86.63 (13.24) (*n* = 24)	87.50 (26.49) (*n* = 15)	82.59 (15.75) (*n* = 8)	97.50 (7.07) (*n* = 8)	89.85 (14.62) (*n* = 18)	78.01 (31.03) (*n* = 12)	80.95 (21.10) (*n* = 5)

For percentage of fulfilled intentions, the number of participants who reported at least one intention in the particular specificity category is reported

Next, age differences in remembering real-life self-assigned PM tasks were examined for all the nominated intentions and as a function of temporal specificity. Results of a one-way ANOVA for all nominated intentions showed that young (*M* = 81.55; SD = 10.93) and older participants (*M* = 85.01; SD = 13.78) did not differ significantly in the percentage of correctly performed intentions, *F* (1, 43) = .88, *p* = .354, $\eta _{{\text{p}}}^{2}$ = .02. Considering real-life PM performance as a function of temporal specificity, separate ANOVAs were run comparing the percentage of fulfilled intentions in young and older adults for each specificity category (i.e., exact date and time, time-window, specific day, deadline).^4^ To avoid an inflated type I error rate, the alpha level was set at .013 according to the number of performed tests (i.e., .05/4 = .013). There was a trend for better performance in the older adults for self-assigned PM tasks with an exact date and time reported during encoding with a large effect size, *F* (1, 20) = 5.06, *p* = .036, $\eta _{{\text{p}}}^{2}$ = .20 (see Table 6). No other comparisons approached significance, all *F*s < 1.

### The role of perceived importance in naturalistic and self-assigned PM tasks

Perceived importance was measured for each naturalistic PM task and each self-assigned intention during the first three study days by asking participants how important they rated each of them using a three-point Likert-scale (1 = less important, 2 = important, 3 = very important). These ratings were then added and divided by the number of tasks/intentions to obtain the average importance rating for each task type. Table 7 (upper panel) shows the mean importance ratings and the results of one-way between-subjects ANOVAs for naturalistic PM tasks (both event- and time-based) and for self-assigned tasks overall and with the date and time specified at encoding. No significant age effects were obtained (all *F*s <2.62), except for event-based naturalistic PM tasks, which were rated as more important by older adults (*M* = 2.14, SD = .48) than young participants (*M* = 1.74, SD = .51).

**Table 7:** Mean importance ratings and reminder use (standard deviations) in young and older adults for event- and time-based naturalistic PM tasks, self-assigned intentions overall, self-assigned intentions with date and time specified, and results of one-way ANOVAs

PM Task	Age group				
	Young	Old	*F*	*p*	$\eta _{{\text{p}}}^{2}$
	*M* (SD)	*M* (SD)			
Importance					
Naturalistic event-based	1.74 (.51)	2.14 (.48)	7.04	.011	.14
Naturalistic time-based	1.93 (.55)	2.18 (.39)	2.62	.113	.056
Self-assigned (overall)	2.11 (.36)	2.15 (.43)	.12	.728	.002
Self-assigned date and time	2.17 (.58)	2.11 (.43)	.06	.806	.003
Reminder use					
Naturalistic event-based	67.86 (51.31)	52.78 (49.92)	.97	.331	.02
Naturalistic time-based	51.92 (47.9)	36.11 (44.74)	1.22	.275	.028
Self-assigned (overall)	12.28 (22.23)	12.67 (28.7)	.003	.956	.000
Self-assigned date and time	13.33 (35.31)	0 (0)	1.12	.301	.05

Importance was measured using a three-point Likert-scale (1 = less important, 2 = important, 3 = very important). Reminder use was measured as the percentage of tasks for which participants reported to have used reminders

### The role of reminder use in naturalistic and self-assigned PM tasks

For each naturalistic PM task and each self-assigned intention during the first three study days, participants were asked if they used a reminder to remember them. The percentage of tasks for which a reminder was used was then calculated for each task type (e.g., if a reminder was used for one of the two event-based naturalistic tasks, reminder use was determined as 50%). Table 7 (lower panel) shows the mean percentages of reminder use in young and older adults as a function of PM task and the results of one-way ANOVAs on these means. No significant age effects emerged for any of the naturalistic PM tasks. Further, no significant age effects were obtained for real-life self-assigned PM tasks either overall or for intentions with the date and time specified at encoding. It is interesting that for self-assigned tasks’ participants reported using reminders for a very small percentage of intentions (between 0 and 13%).

## Discussion

Despite some progress in research on the age–PM-paradox over the past decade, there are several unanswered questions not only in terms of the exact pattern of the paradox for time- and event-based tasks, but also in terms of potential variables that are critical in determining the direction of age effects in and outside the laboratory (Phillips et al., 2008). The aim of the present study was to re-examine the precise pattern of the paradox for time- and event-based PM tasks by studying, for the first time, possible age differences in lab-based, naturalistic and self-generated real-life PM tasks within the same sample of young and older adults. Our main prediction was that a standard pattern of the age–PM-paradox reported in previous studies for time-based tasks would not be obtained for event-based tasks. In other words, while we expected negative age effects in the laboratory, we predicted positive age effects outside the laboratory only for naturalistic time-based but not for event-based tasks. With regards to self-assigned PM tasks, we expected that the pattern of findings would mimic the one obtained for naturalistic PM tasks with no age effects for event- but positive age effects for time-based tasks.

Several novel findings emerged. In line with predictions, no statistically reliable age benefit was found for naturalistic event-based PM tasks, as performance scores of young and older participants did not differ from each other. In contrast, standard (and very large) negative age effects were obtained for laboratory event-based tasks. This finding replicates numerous previous laboratory studies on event-based PM. In addition, the pattern of negative age effects in the lab and the absence of age effects in naturalistic PM tasks is identical with the initial results of Kvavilashvili et al. (2013) and Niedźwieńska and Barzykowski (2012), who also compared the event-based PM performance with lab-based and naturalistic tasks in one sample of young and older participants. This consistency of results on event-based PM tasks across studies using different tasks and materials instills confidence in the conclusion that age benefits found for naturalistic time-based tasks do not generalize to naturalistic event-based tasks.

In contrast to the absence of age effects on naturalistic event-based PM, a significant age benefit was found for naturalistic time-based tasks. This replicates the findings of previous studies on-time-based PM where participants had to remember to carry out the same task several times over an extended period of time (once a day or even several times a day). Our findings extend the results of previous studies by showing that positive age effect was present even when participants were given single rather than repeated time-based tasks over 1- and 3-day delay intervals.

However, somewhat unexpected findings emerged for participants’ own self-assigned PM tasks. First, the examination of the content of nominated intentions did not describe tasks involving event-based cues and most PM tasks were clearly time-based with varying degree of specificity in terms of when the task was to be carried out (see also Ellis, 1988; Ellis & Nimmo-Smith, 1993). Second, when age effects were examined for reported fulfillment of these self-assigned time-based intentions, no age effect emerged in contrast to positive age effects on experimenter-assigned time-based tasks. When PM performance for these tasks was examined as a function of temporal specificity, there was a trend for better performance in older compared to young adults for highly specific intentions with a clear date and time planned for the fulfillment.

Taken together, these findings both replicate and significantly extend existing research on the paradox by providing strong support for the idea that the age benefits with naturalistic and self-assigned PM tasks documented in previous research may have been overestimated due to the dominant use of only those naturalistic time-based PM tasks, in which the time and date are clearly specified. Consequently, we propose to consider redefining the pattern of the age–PM-paradox as consisting of mostly negative age effects in lab-based PM tasks and mostly the absence of negative age effects (rather than age benefits) in naturalistic tasks outside the laboratory. The latter is clearly the case for event-based tasks and also for time-based tasks with less temporal specificity as demonstrated by the findings on participants’ own self-assigned time-based intentions.

Additional findings concerning possible mechanisms underlying the age benefits, observed for some of the naturalistic tasks in the present study, do not support the widespread view that age differences in perceived task importance and reminder use play key roles in this context (cf. Phillips et al., 2008). Indeed, the only age difference in importance ratings for naturalistic tasks was observed for event-based ones, and here, PM performance did not differ between young and older adults. No age differences for perceived importance and reminder use for the time-based naturalistic tasks emerged, although these were the PM tasks in which age benefits were observed. Our results therefore add to the growing number of studies suggesting that age differences in reminder use cannot explain the age–PM-paradox, although intuitively it seems like a plausible argument (Ihle et al., 2012; Maylor, 1990; Patton & Meit, 1993; Rendell & Craik, 2000; Rendell & Thomson, 1999; West, 1988). For example, Niedźwieńska and Barzykowski (2012) also showed that reminder use did not explain the presence and absence of age benefits in time- and event-based naturalistic tasks, respectively. In addition, while Niedźwieńska and Barzykowski (2012) showed that a higher commitment to perform the naturalistic tasks in the older adults (which can be interpreted as a measure of motivation) was associated with age benefits, this factor did not differ as a function of PM cue type. Therefore, although higher motivation to perform PM tasks in everyday life may be beneficial in general, this factor does not seem to explain the performance differences observed in naturalistic PM as a function of cue type.

Future research should focus on factors that are more closely related to specific challenges of each task type, for example, adhering to a specific target time. One possible candidate could be age differences in the structure and number of routine activities which may be higher in older adults and which may make it easier to implement PM tasks in general and convert time-based into event-based tasks or vice versa (Maylor, 1990; Rendell & Craik, 2000). The trend for a higher number of planned intentions in the young compared to the older adults, observed in the present study, may suggest that young adults are generally busier, which may make a timely execution of additional tasks in one’s everyday life more challenging. In line with this, previous research showed that young adults reported being more stressed in their everyday life compared to older adults (Schnitzspahn et al., 2011) and that higher levels of self-reported stress were negatively related to naturalistic PM (Ihle et al., 2012).

Recent findings by Niedźwieńska et al. (2013) suggest that better PM performance of older adults in naturalistic PM tasks can be partly explained by better planning. More precisely, older adults displayed a better temporal organization of their self-assigned real-life PM tasks and specified more clearly when they would be fulfilled. These results raise the possibility that older adults may be more likely than young adults to use detailed and effective plans to implement their intentions, especially for time-based tasks. In the present study, older adults did not form more self-assigned PM tasks with highly defined temporal cues than young adults, but they performed these intentions especially well with a trend for better performance compared with the young adults. This finding further supports the idea that older adults benefit from precisely planned intentions in their everyday life and that cue specificity may be an important factor underlying the age benefit usually found for time-based PM tasks outside of the laboratory.

While young adults seem to struggle to perform naturalistic time-based PM tasks at specific time points predefined by the experimenter, overall, there was no age effect on self-assigned time-based task performance. Given that young and older adults mainly indicated to have set themselves intentions with certain time-windows instead of intentions that had to be executed at a precise time and date, young adults had more flexibility performing these tasks compared to the naturalistic time-based PM tasks. This greater flexibility may have enabled young adults to fulfill their intentions more easily according to their (rather vague) plans despite potentially higher stress levels and poorer time management skills.

At first glance, it may seem surprising that we observed age impairments in event-based, but not time-based laboratory PM tasks. Time-based tasks lack an external event that indicates the appropriate moment to initiate the intended action and therefore are assumed to require more self-initiated processing (e.g., monitoring of the time; Einstein et al., 1995; Kvavilashvili & Fisher, 2007). However, in line with our findings, the meta-analysis by Henry et al. (2004) could not confirm the widespread assumption that time-based PM tasks produce larger age effects than event-based ones for lab-based and naturalistic tasks. At this point, it is difficult to offer a theoretical explanation of these findings, as research on-time-based PM is limited and specific theories are missing. Although the influential multiprocess model focuses on event-based PM, McDaniel and Einstein (2000) indicated that aspects of this theory may be relevant to time-based tasks as well. For example, time-based PM may also depend on spontaneous or controlled processes as a function of task characteristics, such as ongoing task difficulty, the time-window for responding, the ease with which the elapsed time can be monitored, etc.

While the ongoing laboratory activities used in the present study were attentionally demanding (i.e., different memory and intelligence tests and questionnaires), they also offered opportunities for breaks between different tasks that might have been used for rehearsal and clock checking. Further, they did not require any motoric coordination for pressing different keys on the keyboard (i.e., to carry out the ongoing task, PM task and to check the clock), which older participants sometimes struggle with when completing computerized PM tasks. It is noteworthy that Niedźwieńska and Barzykowski (2012), who used a similar time-based PM task in the lab with comparable ongoing activities (i.e., performing different memory and intelligence tasks and rating restaurant descriptions), also did not find any age difference. Finally, in event-based tasks, the appearance of a target event is unpredictable while in laboratory time-based tasks, estimating the elapsed time is under participants’ control as they can decide when to check the time. In line with this argument, several studies have shown that event-based tasks had more detrimental effects on performance in the ongoing task than time-based tasks (e.g., Park et al., 1997; Trawley, Stephens, Rendell, & Groeger, 2017).

In sum, these findings cast some doubts on the assumption that time-based tasks are always attentionally more demanding than event-based tasks (see also Kvavilashvili & Fisher, 2007), and suggest that more targeted investigation of this question is necessary. Conceptually, these investigations could be based on a recent extension of the multiprocess model of PM (Scullin et al., 2013; Shelton & Scullin, 2017), as it offers a broader theoretical framework that can be applied to both event- and time-based tasks. This dynamic multiprocess view suggests an interplay between spontaneous and controlled processes within the same PM task. Future research should examine how the type of PM task and the setting in which it is completed may moderate the suggested dynamic interaction between bottom-up, spontaneous and top-down, controlled processes supporting time-based PM.

The distinction between time- and event-based tasks is well established in PM research. The current finding that the pattern of the age–PM-paradox needs to be reconsidered as a function of the cue type (i.e., time- vs. event-based) underlines the usefulness of this distinction. Indeed, both task types seem to measure related, but distinct abilities that can be differentially influenced by age and thus should be considered separately. However, the fact that the participants in the present study reported hardly any self-assigned intentions related to a specific environmental cue, event or situation, and instead planned the fulfillment of their intentions in relation to a more or less specific point in time depicts an interesting finding in itself and is in line with a previous diary study (Holbrook & Dismukes, 2009) that also reported a remarkable absence of event-based PM tasks and a clear prevalence of time-based PM tasks in daily life. These findings might question the usefulness of the distinction into time- and event-based PM tasks in everyday life.^5^ In line with this observation, Dismukes (2012) suggested that the categories of event-based and time-based PM do not capture the range of situations in which individuals perform intended actions in their everyday lives and therefore a clear categorization can be very difficult. More research is needed to verify which types of PM tasks people actually face in their everyday lives. In any case, the marked dominance of self-assigned time-based intentions in the present study underlines the importance of this task type from an applied perspective. Accordingly, PM research in and outside of the laboratory should focus more on-time-based PM and define different variants within this task type depending on temporal specificity, regularity or length of delay. In addition, future research should also focus on-time-based tasks that come up during the day rather than being intended well in advance (e.g., when calling someone and being asked to call back in an hour). So far, only Rendell and Craik (2000, study 2) investigated such ‘crop up’ naturalistic time-based tasks (referred to as ‘time-check’ tasks), and found no significant age effect between young and old–old participants, but both groups performed reliably worse than young-old participants. The latter finding also underscores the importance of having young–old and old–old participants in studies on the age–PM-paradox, which was one of the limitations of the present study.

Another possible limitation involved the design of naturalistic event-based PM tasks, as the possibility that some participants did not perform the tasks just because the opportunity to encounter the PM cue (i.e., pass a post box or see a bus) was missing, cannot be ruled out completely. However, all participants lived in a big city with a high number of post boxes and buses in their everyday environment, and stated going out on a daily basis, which should have reduced the chances of this occurring, and none of the participants reported informally that this was the cause of their forgetting.

Given that older adults in our study owned a mobile phone and were proficient in sending text messages, it is possible that we recruited a sample of highly functioning older adults who were not representative of the general population, especially of adults in their late 70 s and 80 s. However, the use of mobile phones is increasingly becoming a norm across the world in both young and older adults. Moreover, older participants in the present study were showing large cognitive declines in the standard laboratory tasks of fluid intelligence and episodic memory (see Table 1), but these deficits did not seem to influence their performance on naturalistic and self-assigned PM tasks. Old–old adults (over 75 years of age) would probably show stronger and more consistent PM impairments in the laboratory than in the present sample, while they might still perform comparably to young adults in the field (e.g., Kvavilashvili et al., 2013; Rendell & Craik, 2000; Rendell & Thomson, 1999).

In summary, results of the present study provide important insights into the precise pattern of the age–PM-paradox. In particular, the results showed that the previously documented superiority of older adults in remembering naturalistic PM tasks could be due to the use of time-based tasks with high temporal specificity, and that positive age effects outside the laboratory disappear when event-based tasks are used (cf. Niedźwieńska & Barzykowski, 2012). This differential pattern of age effects in naturalistic event- and time-based PM tasks calls for more targeted and fine-grained investigation of potential underlying mechanisms of these age effects in future studies.

The finding that there may be no age effects outside the laboratory for naturalistic and self-assigned real-life PM tasks, does not reduce the importance of the re-defined age–PM-paradox for research in cognitive aging and its practical implications. Indeed, the finding that older adults can function cognitively as well as young adults in everyday life while displaying substantial deficits in laboratory PM tasks and other cognitive tasks measuring speed of processing, working memory and long-term memory (Park, Polk, Mikels, Taylor, & Marshuetz, 2001), is important not only theoretically, but also practically due to popular culture and prevailing beliefs that cognitive functions significantly deteriorate in old age. Although the age–PM-paradox has been suggested to be unique to PM and not extend to retrospective memory in general (Phillips et al., 2008), latest findings reported in the retrospective memory literature on flashbulb memories (Kvavilashvili, Mirani, Schlagman, Erskine, & Kornbrot, 2010), memories for incidental stimuli in the environment (Qin et al., 2014) and involuntary autobiographical memories (Berntsen et al., 2017; Kvavilashvili, Niedźwieńska, & Kliegel, 2016), to name the few, suggest that the absence of age effects for naturalistic tasks may be more widespread than previously thought.

### Availability of data and material

The dataset generated and analysed during the current study is available from the corresponding author upon reasonable request.

## Footnotes

1 Note that in the Rendell and Craik (2000) study, only old participants (young-old and old-old) completed both laboratory and naturalistic PM tasks (in study 1 and 2, respectively), while separate groups of young adults were recruited for study 1 and 2.

2 It is interesting that the categories of ‘exact date and time’, ‘time-window’ and ‘entire day’ are identical to the categories in Ellis and Nimmo-Smith’s (1993) classification of time-based tasks as ‘pulses’, ‘intermediates’ and ‘steps’, respectively.

3 Analyzing performance separately for the two naturalistic event-based tasks (i.e. bus and post box task) showed the same pattern of results (i.e., no age differences in performance).

4 The data set did not allow running a mixed ANOVA, because the dependent variable was not a repeated measure in the usual sense. Thus, participants were not asked to form intentions with cues of various specificity. Instead, they reported all their daily intentions and indicated if they planned to carry them out in relation to an event, time or date. As a result, not all participants reported each of the four different types of intentions (see Table 5).

5 It is important to point out that the absence of event-based intentions in participants’ self-assigned PM tasks was not due to the assessment method (i.e., by asking participants to indicate a possible time frame for nominated intentions). Indeed, participants could describe an event-based task (e.g., pass on a message to a friend) and then indicate an approximate time frame when this event could be encountered (e.g., tomorrow). However, there was absence of such events in participants’ descriptions, indicating that in everyday life perhaps people form event-based intentions less frequently than time-based intentions. For example, an intention to pass on a message was only described by one participant on one occasion.
